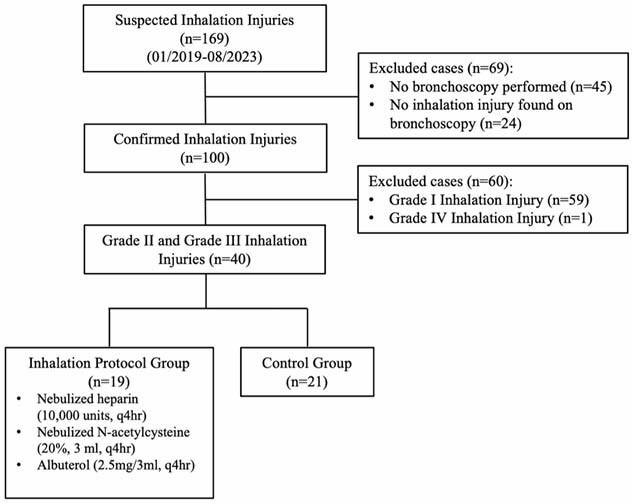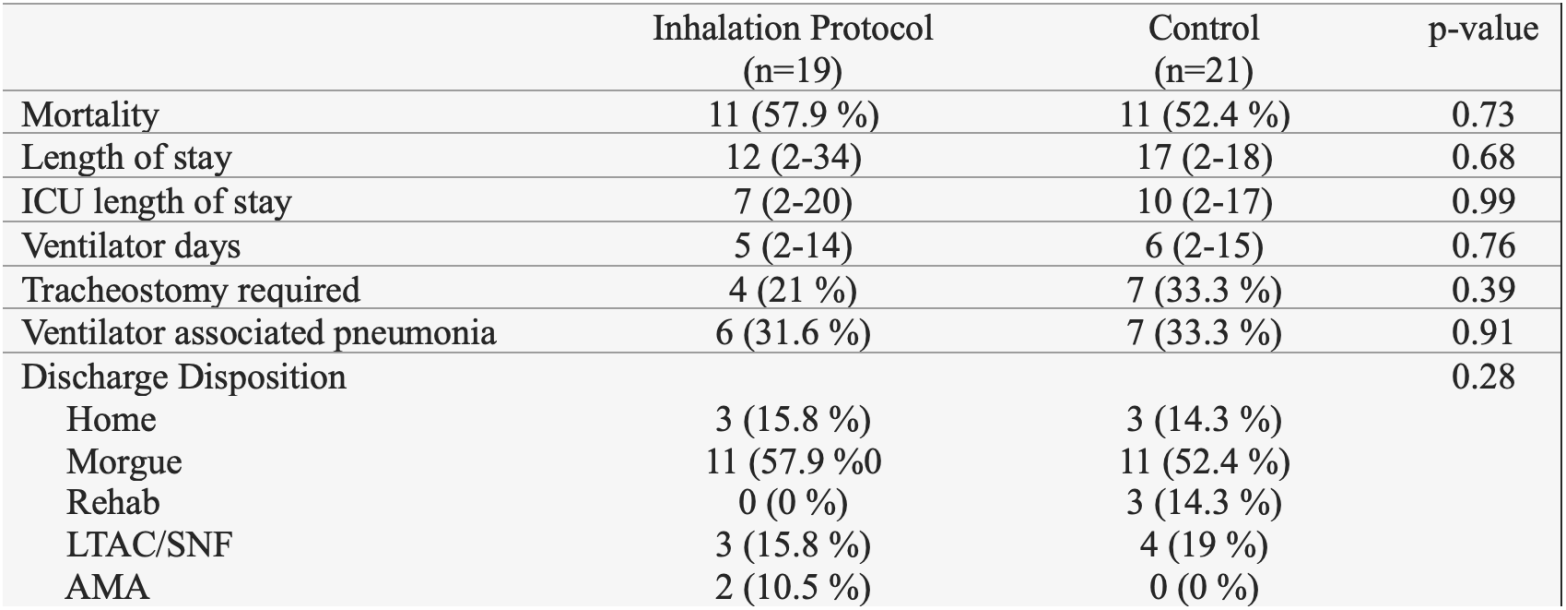# 888 Nebulized Heparin and N-Acetylcysteine Do Not Improve Outcomes of Intubated Burn Patients with Inhalation Injuries

**DOI:** 10.1093/jbcr/iraf019.419

**Published:** 2025-04-01

**Authors:** Brittany Sims, Will Risinger, Victoria Hammond, Chinweotuto Uma, Keeven David

**Affiliations:** University of Louisville; University of Louisville; University of Louisville; University of Louisville; University of Louisville

## Abstract

**Introduction:**

Inhalational injury significantly alters the clinical course of burn patients. In addition to increasing the volume of fluid required for adequate resuscitation, inhalation injury places patients at risk of prolonged mechanical ventilation, pneumonia, and acute respiratory distress syndrome. To combat the deleterious effects of inhalational trauma, therapeutic strategies consisting of nebulized medications have been used. Nebulized heparin attenuates pulmonary edema and reduces formation of intraluminal fibrin. NAC provides anti-inflammatory benefits in addition to a mucolytic. Albuterol functions as a bronchodilator. We sought to perform a pilot study at our institution. We hypothesized that adjunctive heparin and NAC would decrease ventilator dependence and in-hospital mortality.

**Methods:**

Individual cases were reviewed and only those confirmed by fiberoptic bronchoscopy were included. The final study population consisted of patients with Grade II and III injury patterns. The protocol consists of 10,000 units of nebulized heparin, 3 ml of 20% nebulized NAC, and 3 ml of 0.083% nebulized albuterol all given every 4 hours for either 7 days or until extubation. Demographics, injury characteristics, and hospital outcomes were compared between the treatment and control groups. Wilcoxson rank-sum test and Pearson’s chi-square statistic were used for numerical and categorical variables respectively. For all analyses, p < 0.05 was considered statistically significant.

**Results:**

40% of initial cohort suffered from Grade II or Grade III injury patterns and were included in the final study population. Of these, 47.5% were treated with the inhalation injury protocol, with the remaining 52.5% serving as historical controls. Patients receiving adjunctive nebulized therapies had similar outcomes compared to historical controls. Hospital length of stay as well as time in the intensive care unit did not differ. Likewise, there was no statistically significant decrease in days of ventilator dependence. Ultimately, mortality rates did not differ for patients receiving nebulized therapies.

**Conclusions:**

The results of our pilot study demonstrated no difference in ventilator dependence or in-hospital mortality with the utilization of nebulized heparin, NAC, and albuterol in adult burn patients suffering inhalational trauma. Despite disagreeing with the findings of previous studies, these results should not deter the current use of nebulized therapies which have been deemed “appropriate” for use in the management of inhalation injury by an international panel. However, these results stress the need for a vigorous, multicenter effort to perform a randomized control trial.

**Applicability of Research to Practice:**

This study is directly applicable to bedside practice and management of intubated patients with inhalational burn injuries in an attempt to improve patient outcomes with nebulized therapies

**Funding for the Study:**

N/A